# MiR-766 induces p53 accumulation and G2/M arrest by directly targeting MDM4

**DOI:** 10.18632/oncotarget.15530

**Published:** 2017-02-20

**Authors:** Qingqing Wang, Luke A. Selth, David F. Callen

**Affiliations:** ^1^ Breast Cancer Genetics Group, Centre for Personalised Cancer Medicine, School of Medicine, University of Adelaide, Adelaide, South Australia; ^2^ Dame Roma Mitchell Cancer Research Laboratories, School of Medicine, University of Adelaide, Adelaide, South Australia; ^3^ Freemasons Foundation Centre for Men’s Health, Discipline of Medicine, University of Adelaide, Adelaide, South Australia

**Keywords:** MicroRNA, MDM4, p53, cancer, cell cycle

## Abstract

p53, a transcription factor that participates in multiple cellular functions, is considered the most important tumor suppressor. Previous evidence suggests that post-transcriptional deregulation of p53 by microRNAs contributes to tumorigenesis, tumor progression and therapeutic resistance. In the present study, we found that the microRNA miR-766 was aberrantly expressed in breast cancer, and that over-expression of miR-766 caused accumulation of wild-type p53 protein in multiple cancer cell lines. Supporting its role in the p53 signalling pathway, miR-766 decreased cell proliferation and colony formation in several cancer cell lines, and cell cycle analyses revealed that miR-766 causes G2 arrest. At a mechanistic level, we demonstrate that miR-766 enhances p53 signalling by directly targeting MDM4, an oncogene and negative regulator of p53. Analysis of clinical genomic data from multiple cancer types supports the relevance of miR-766 in p53 signalling. Collectively, our study demonstrates that miR-766 can function as a novel tumor suppressor by enhancing p53 signalling.

## INTRODUCTION

p53 is a nuclear phosphoprotein encoded by the *TP53* gene [1] that is widely recognized to be the most important tumor suppressor in the cell. As a transcription factor, p53 responds to various stresses and activates multiple pathways including apoptosis, cell cycle arrest and DNA repair – hence its designation as the “guardian of the genome”. Recent studies reveal diverse additional functions of p53, including functions in cell stemness [2], epithelial–mesenchymal transition (EMT) and tumor metastasis [3, 4], tumor angiogenesis [5] and cellular senescence [6]. Given its critical role as a tumor suppressor, it is not surprising that *TP53* is frequently mutated in cancer. Indeed, mutation of this gene occurs in over 50% of cancers [7], enabling malignant cells to escape wild-type p53-dependent growth inhibition and cell death.

The expression and activity of p53 is tightly regulated by, amongst other mechanisms, ubiquitination, phosphorylation and nuclear/cytoplasmic translocation. Two key negative regulators of p53 are MDM2 and MDM4. MDM2 is a specific E3 ligase for p53, promoting its polyubiquitination and subsequent degradation by the proteasome [8, 9]. MDM4, through its BOX1 domain, binds directly to p53 and inhibits its activity [10, 11]. MDM4 also interacts with MDM2 directly [12, 13] to enhance MDM2-mediated ubiquitination and p53 degradation [14].

MicroRNAs (miRNAs), small non-coding RNAs that negatively regulate gene expression by binding to complementary sequences in their targets [15], have been shown to play an important role in the post-transcriptional regulation of p53. For example, several miRNAs and miRNA families directly regulate p53, including miR-125b [16], miR-504 [17], miR-380-5p [18], miR-25 and miR-30d [19]. Other miRNAs can upregulate p53 expression and activity by targeting MDM2 and MDM4. For example, miR-34a directly targets MDM4 by binding to a site in its open reading frame (ORF) [20, 21]. Interestingly, miR-34a is also a downstream target of p53 [22–24], and therefore represents a positive feedback loop for p53 through MDM4 inhibition. Similarly, miR-605, miR-192 and miR-215, which directly target MDM2, are also downstream targets of p53 [25]. Moreover, miR-339-5p [26] and miR-661 [27] also promote p53 activity and stabilities by targeting MDM2 and/or MDM4, while miR-122 [28], miR-885-5p [29] and miR-542-3p stabilise p53 in cancer cells by disrupting MDM2-mediated p53 degradation [30].

MiR-766-3p (miR-766) is a microRNA residing within an intron of the *SEP6* gene. Several studies indicated that miR-766 expression was highly expressed in cutaneous squamous cell carcinoma biopsies [31], lung adenocarcinoma (LUAD) [32] and acute promyelocytic leukemia cells [33]. In this present study, we analyzed small RNA sequencing data from The Cancer Genome Atlas (TCGA) and identified miR-766 as a putative post-transcriptional regulator of p53. We demonstrated that miR-766 stabilised p53 by targeting the 3′UTR of MDM4, leading to repression of cell growth and cell cycle arrest in cancer cells and enhancing the p53 signalling pathway. Overall, our study indicates that miR-766 is a new and important regulator of p53-dependent tumor suppression.

## RESULTS

### MiR-766 induces wild-type p53 protein accumulation and cell growth repression

We analyzed small RNA deep sequencing data of breast cancer tumors expressing wild-type p53 (228 tumors) or missense p53 (57 tumors) downloaded from TCGA. Compared to tumors with wild-type p53, miR-766 was elevated in mutant p53 tumor samples (Supplementary Figure 1A). More data was collected from TCGA across different cancer types (including hepatocellular carcinoma, lung squamous cell carcinoma, lung adenocarcinoma, colon adenocarcinoma, stomach adenocarcinoma and ovarian serous cystadenocarcinoma), and a trend of increased miR-766 expression in p53 mutant groups was found (Supplementary Figure 1B). Given that highly expressed wild-type p53 induces cell proliferation arrest or cell programmed death, cancer cells tend to suppress factors that activate it [34]. We hypothesized that the down-regulation of miR-766 in wild-type p53 tumors compared to mutant p53 may indicate a potential connection between miR-766 and p53 function.

**Figure 1:**
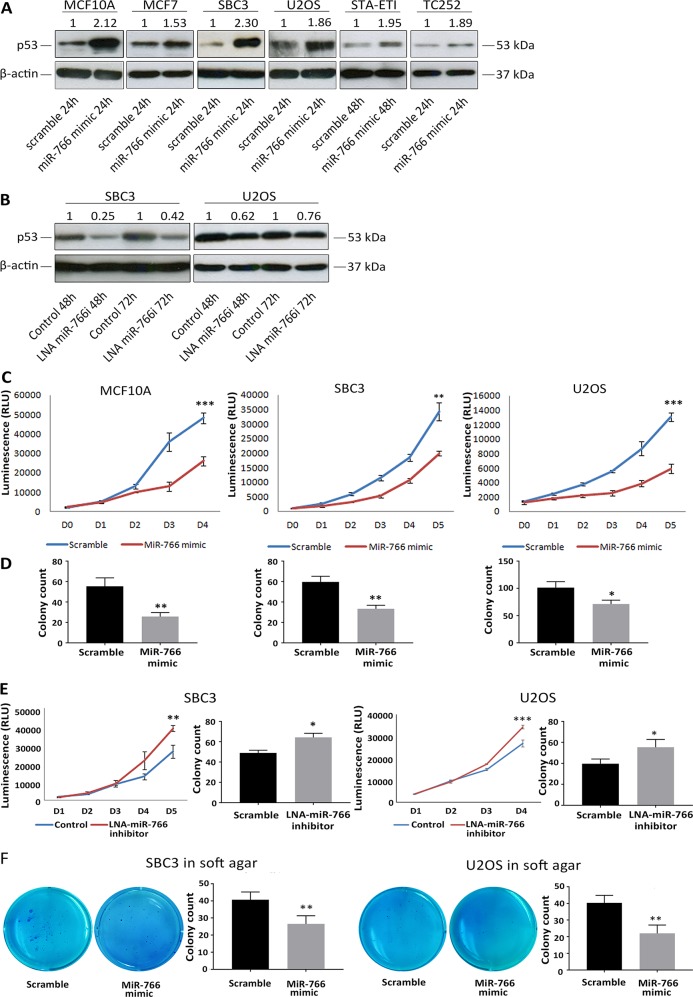
MiR-766 upregulates p53 expression and blocks cancer cell growth **(A)** Whole protein lysates were collected 24 and 48 hours following transfection with miR-766 mimic or a “scramble” control miRNA mimic. p53 protein levels were detected by Western blotting (β-Actin was used as a loading control). **(B)** Whole protein lysates were collected 24 and 48 hours following LNA miR-766 inhibitor or LNA control transfection. p53 protein levels were detected by Western blotting (β-Actin was used as a loading control). **(C)** Cell proliferation was determined by Cell-TiterGlo Assay from in U2OS, SBC3 and MCF10A cells following transfection with miR-766 mimic or a “scramble” control miRNA mimic. **(D)** Colony formation assay was performed on U2OS, SBC3, MCF7 and MCF10A cells following transfection with miR-766 mimic or a “scramble” control miRNA mimic. **(E)** Cell proliferation was determined by Cell-TiterGlo Assay in SBC3 cells following transfection with LNA miR-766-inhibitor or LNA control. Colony formation assay was performed on SBC3 cells following transfection with LNA miR-766-inhibitor or LNA control. **(F)** Soft agar colony formation assay was performed using miR-766 mimic transfected SBC3 and U2OS cells in 0.7%-0.3% agar gel and incubated for 3 weeks. For all assays, p values (* p<0.05, **p<0.01, *** p<0.001) were determined by Student’s t-test.

To test this hypothesis, we ectopically over-expressed miR-766 in a panel of wild-type p53 cancer cell lines derived from breast cancer, lung cancer and sarcoma (Supplementary Figure 2) to observe its effect on p53 levels. In all cell lines examined, we observed increased p53 protein levels following transfection of miR-766 mimic (Figure 1A). Conversely, inhibition of miR-766 using an LNA inhibitor reduced p53 protein in SBC3 and U2OS cells, further supporting the concept that this miRNA maintains p53 expression (Figure 1B). To determine if these effects were transcriptional or post-transcriptional, p53 mRNA was measured following miR-766 over-expression in MCF10A, SBC3 and U2OS cells. No significant change in p53 mRNA was observed (Supplementary Figure 3), suggesting that p53 protein levels were increased by miR-766 at a post-transcriptional level.

We next asked whether the miR-766-mediated increase in p53 protein was associated with enhanced p53 cellular activity. Cell proliferation assays demonstrated that the growth of MCF10A, SBC3 and U2OS cells was significantly suppressed after ectopic over-expression of miR-766 (Figure 1C). Moreover, using a colony formation assay, we found that miR-766 decreased clonogenicity in MCF10A, SBC3 and U2OS cells (Figure 1D). Conversely, inhibition of miR-766 using an LNA inhibitor increased cell proliferation and colony formation of SBC3 and U2OS cells (Figure 1E). A soft agar colony formation assay was used to further investigate the anti-growth capacity of miR-766. As shown in Figure 1F, the ability of SBC3 and U2OS cell lines to generate spheres was significantly impaired after miR-766 over expression in both S. Collectively, these data reveal that miR-766 increases wild-type p53 levels, resulting in reduced cell proliferation and colony formation.

### MiR-766 promotes G2/M arrest in U2OS and SBC3 cells

**Figure 2:**
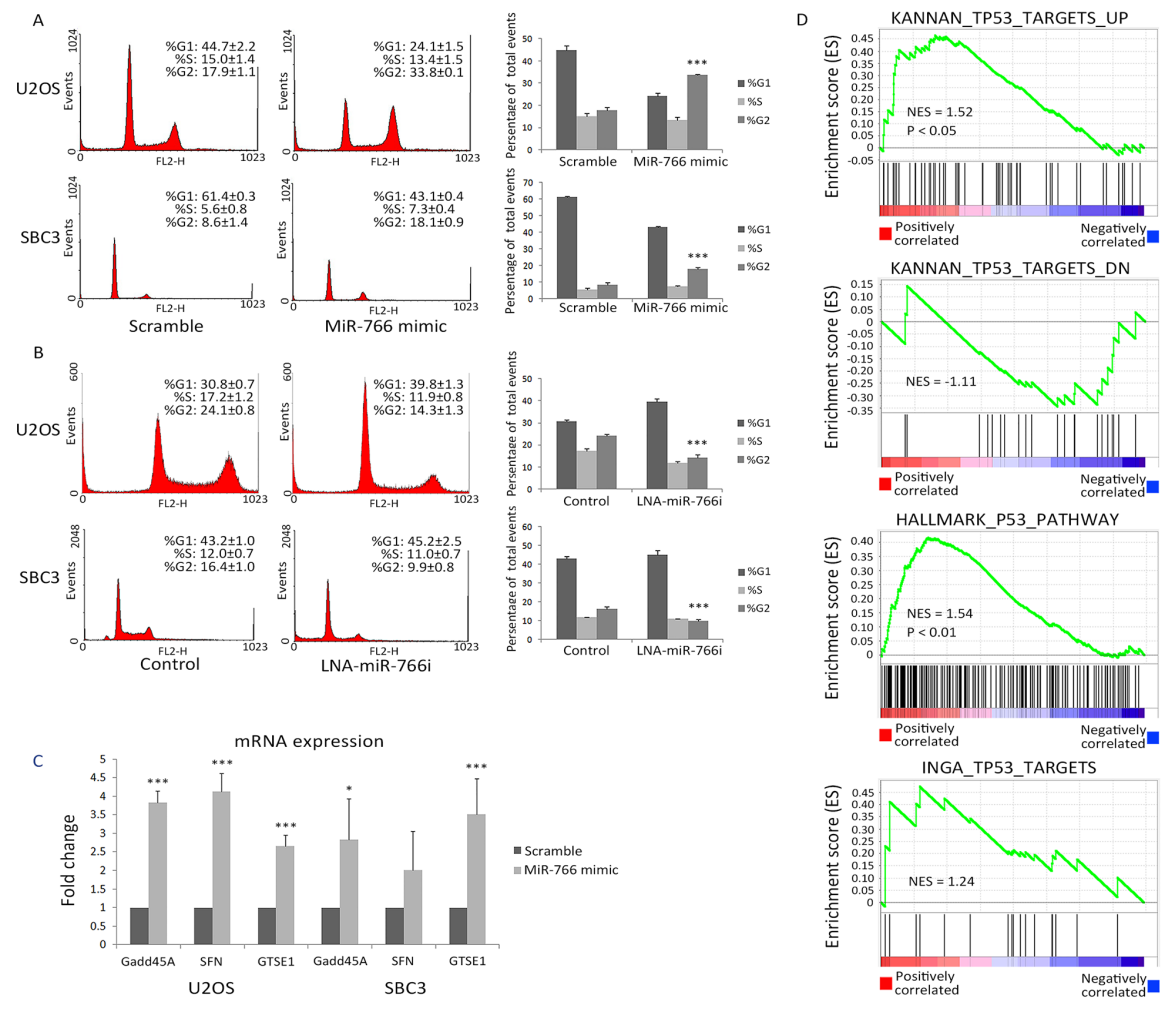
miR-766 is positively correlated with active p53 signalling **(A-B)** Cell cycle profiles were analyzed through PI staining in U2OS and SBC3 48 hours after **(A)** miR-766 over-expression and **(B)** miR-766 inhibition. **(C)** Total RNA was collected from SBC3 and U2OS cells following transfection with miR-766 mimic or a “scramble” control miRNA mimic. *GADD45A*, *SFN* and *GSTE1* mRNA levels were detected by qRT-PCR (normalized using *GAPDH*). Results are the average of three independent experiments. p values (* p<0.05, *** p<0.001) were determined by Student’s t-test. **(D)** MiR-766 is positively correlated with p53 signalling in lung cancer, as determined by gene set enrichment analysis. The correlation between expression levels of miR-375 and 20,531 genes was calculated using matched miRNA and mRNA data from 102 lung tumors. Genes were subsequently ranked according to Pearson correlation coefficient (r) value (shown by a heat map), and GSEA (Preranked analysis) was implemented using the Broad Institute’s public GenePattern server, using default parameters.

To further explore the biological function of miR-766, flow cytometry was used to analyze its impact on cell cycle regulation. We observed a significantly increased proportion of cells in G2 phase 48 hours after miR-766 mimic transfection in U2OS and SBC3 cells (Figure 2A). Specifically, the proportion of cells blocked at the G2/M checkpoint increased from 17.9% to 33.8% in U2OS cells and 8.6% to 18.1% in SBC3 cells. Conversely, a decrease of G2 phase cells was observed in U2OS (24.1% to 14.3%) and SBC3 (16.4% to 9.9%) cells after miR-766 inhibition (Figure 2B).

To detect molecular alterations in the p53 pathway associated with changes in miR-766 expression, we characterized the mRNA levels of *GADD45A*, *SFN* and *GEST1* genes (as these genes are p53 downstream targets involved in cell cycle regulation) following transfection of miR-766 mimic. As expected, all of these genes were significantly induced by miR-766 (Figure 2C), which is consistent with observed increased expression of p53 and G2/M phase.

To explore the association between miR-766 and p53 signalling in clinical samples, we utilized gene set enrichment analysis (GSEA). By analysing lung cancer samples from TCGA, we identified a positive association between miR-766 expression and genes upregulated by p53 in 3 distinct gene sets, and a negative association between miR-766 and genes down-regulated by p53 in one gene set (Figure 2D). These associations further support the concept that miR-766 enhances p53 activity in cancer.

### MDM4 is a direct target of miR-766

**Figure 3:**
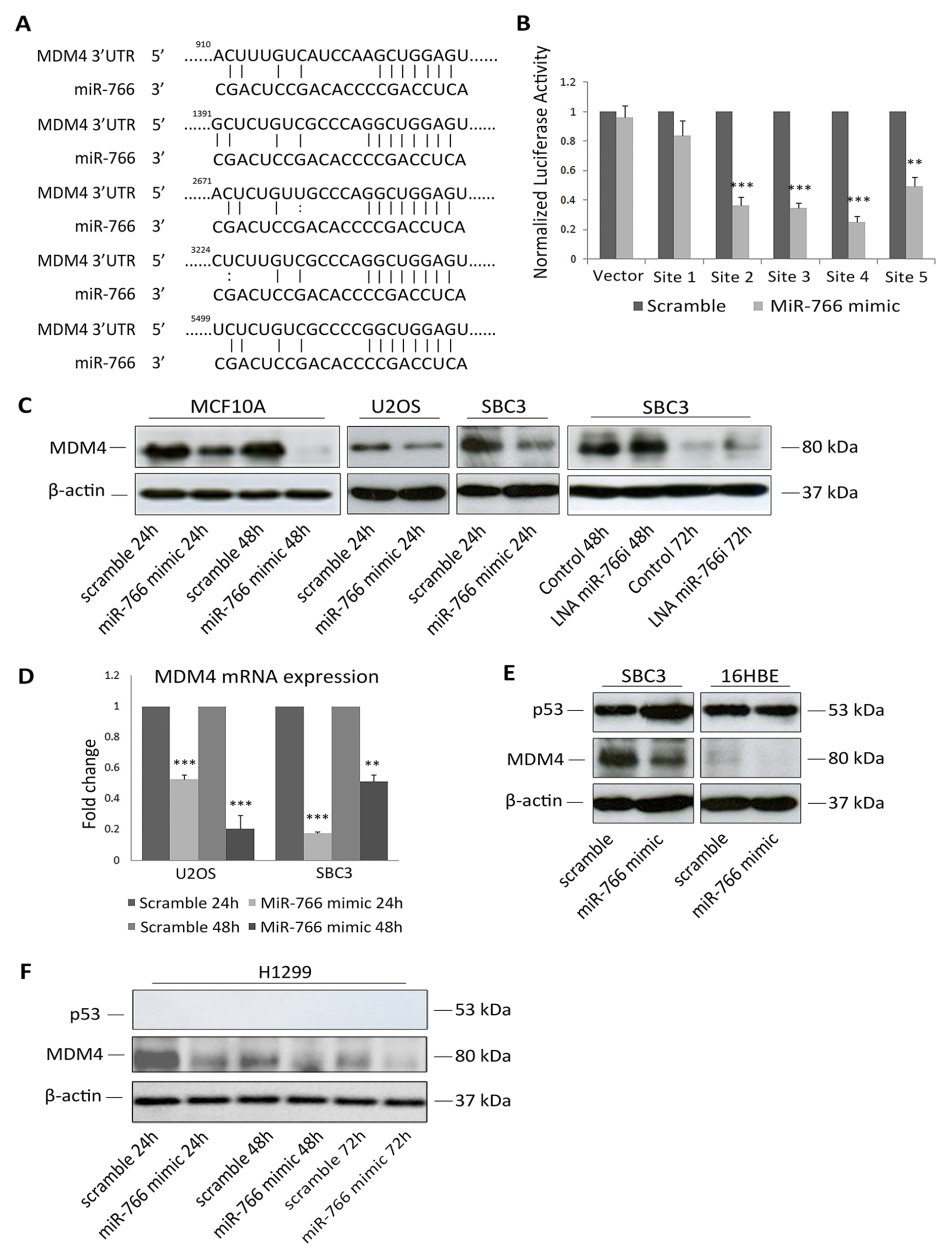
MDM4 is a direct target of miR-766 **(A)** Putative miR-766-binding sequence in the 3′UTR of *MDM4* mRNA. **(B)** Luciferase reporter assays 48 h after transfection with miR-766 mimics and reporter plasmids. Results are the average of 3 independent experiments. p values (* p<0.05, *** p<0.001) were determined by determined by unpaired *t* tests. **(C)** MCF10A, U2OS, SBC cells were transfected with miR-766 mimic or LNA miR-766-inhibitor and control. Whole protein lysates were collected 24 and 48 hours after transfection and p53 and MDM4 protein levels were detected by Western blotting (β-Actin was used as a loading control). **(D)** Total RNA was collected after miR-766-mimic transfection in U2OS and SBC3 cells. *MDM4* mRNA levels were detected by qRT-PCR (normalized using *GAPDH*). Results are the average of 3 independent experiments. p values (* p<0.05, *** p<0.001) were determined by Student’s t-test. **(E-F)** SBC3 and 16HBE **(E)** and H1299 **(F)** cells were transfected with miR-766 mimic and a scramble control. Whole protein lysates were collected 24, 48 and 72 hours after transfection and p53 and MDM4 protein levels were detected by Western blotting (β-Actin and a-tubulin were used as loading controls).

Since target genes of miRNAs are expected to be downregulated, we speculated that miR-766 may directly target one or more negative regulators of p53. The online miRNA target prediction tool miRDB.org lists 5 putative binding sites for miR-766 in the 3′UTR of *MDM4* mRNA (Figure 3A). To validate this potential interaction, sequences carrying the predicted miR-766 recognition sites were cloned downstream of luciferase and used for reporter assays. Sites 2, 3 and 4 were strongly repressed by miR-766, site 5 weakly, and site 1 was not regulated (Figure 3B). Having confirmed a direct interaction between miR-766 and sites in the *MDM4* 3′UTR, we subsequently transfected cells with miR-766 mimic and measured MDM4 expression. As shown in Figure 3C and 3D, miR-766 over-expression reduced both MDM4 protein and mRNA levels 48 hours in MCF10A, SBC3 and U2OS cells. Conversely, miR-766 inhibition with an LNA increased MDM4 protein (Figure 3C). To confirm the specificity of MDM4 targeting by miR-766, an unrelated miRNA, miR-375, was used as an additional negative control (Supplementary Figure 4). Finally, to further validate the activity of miR-766, we assessed its effect on the expression of a previously reported target gene, *BAX* [33]: as expected, miR-766 caused a significant reduction of Bax protein (Supplementary Figure 5).

To further assess the relevance of the miR-766-MDM4 interaction in p53 signalling, we employed the 16HBE lung epithelial cell line, which expresses wild-type p53 but very low levels of MDM4 compared with SBC3 (Figure 3E). Although MDM4 was down-regulated by miR-766 in this line, there was no effect on p53, which can be explained by the very low basal levels of MDM4, while p53 was strongly increased in SBC3 following MDM4 inhibition. Considering the negative feedback regulation of the p53-MDM2-MDM4 axis [35], we also examined miR-766-mediated targeting of MDM4 in a p53-negative line, H1299, to exclude the possibility that the change in MDM4 after miR-766 over expression was a result of p53 accumulation (Figure 3F). Although MDM4 protein was low in general in this model system, it was further reduced by miR-766 transfection, verifying the direct link between these two factors.

**Figure 4:**
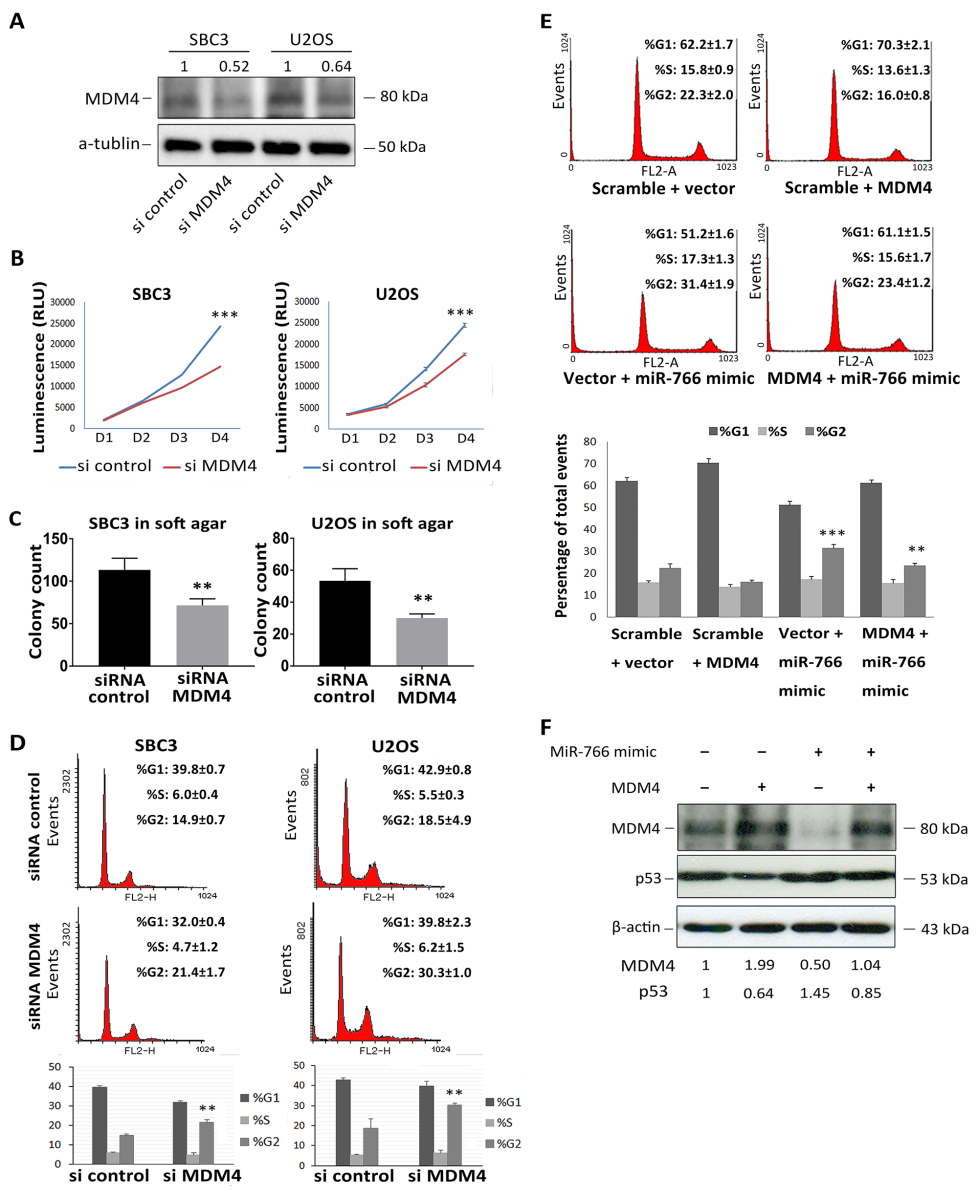
*MDM4* is a biologically relevant target of miR-766 **(A-D)** SBC3 and U2OS cells were transfected with MDM4 siRNA, **(A)** whole protein lysates were collected 48 hours after transfection, MDM4 protein levels were detected by Western blotting (a-tubulin used as loading control); **(B)** Cell proliferation was determined by Cell-TiterGlo Assay following transfection; **(C)** transfected SBC3 and U2OS cells were plated in 0.7%-0.3% soft agar and incubated for 3 weeks; **(D)** Cell cycle profiles were analyzed through PI staining 48 hours after transfection. Results are the average of three independent experiments. p values (**P<0.01; *** *p<0.001)* were determined by Student’s t-test. **(E)** SBC3 cellswere co-transfected with miR-766 mimic and a MDM4 over-expression vector or a control vector. Cell cycle profiles were analyzed through PI staining 48 hours after transfection. Results are the average of three independent experiments. p values (**P<0.01; *** *p<0.001)* were determined by Student’s t-test. **(F)** Whole protein lysates were collected from cells 48 hours after transfection as described in E. p53 and MDM4 protein levels were detected by Western blotting (β-Actin was used as a loading control).

To validate the impact of MDM4 knockdown in our model systems, U2OS and SBC3 cells were transfected with MDM4 siRNA. Consistent with our hypothesis, knockdown of MDM4 (Figure 4A) pheno-copied miR-766 over expression: more specifically, it induced significant cell growth repression (Figure 4B), decreased soft agar clonogenicity (Figure 4C) and caused an increase in G2/M phase cells (Figure 4D).

To further validate the biological relevance of the miR-766-MDM4-p53 regulatory pathway, a vector expressing the MDM4 cDNA without its 3′UTR was constructed. Since expression of MDM4 protein from this vector would be independent of miR-766, it was used in “rescue” experiments. Over-expression of MDM4 reversed both G2/M arrest (Figure 4E) and p53 accumulation (Figure 4F) induced by miR-766 over expression in SBC3 cells. This experiment confirms that MDM4 is likely to be the key target by which miR-766 enhances the p53 signalling pathway.

## DISCUSSION

An increasing number of miRNAs have been recognized to participate in the post-transcriptional regulation of p53 and its downstream pathways. In this study, we have shown for the first time that miR-766 enhances p53 levels and signalling activity by directly targeting the p53 repressor, MDM4. By targeting MDM4, miR-766 enhances p53-mediated cell cycle arrest and repression of proliferation.

Cell cycle regulation is one of the core functions of p53. By transactivating downstream targets of p53, including *CDKN1A*, *GADD45A*, *SFN* and *GTSE1*, p53 induces cell cycle arrest, primarily at the G1/S and G2/M check points [36]. MiRNAs have been previously reported to participate in p53-related cell cycle regulation. In our study, *GADD45A*, *SFN* and *GTSE1* were significantly elevated by miR-766. Given that GADD45A [37] and SFN [38] function as inhibitors of G2/M progression, and GTSE1 is a p53-inducible gene that specifically delays the G2/M transition, we hypothesize that these genes are involved in the observed cell cycle arrest mediated by miR-766 over-expression.

While a number of studies describing roles for miR-766 in different cancer types have been published, overall its function in malignancy is poorly characterized. In acute promyelocytic leukemia cells, miR-766 was reported to be down-regulated following arsenic trioxide (As2O3) treatment, resulting in elevation of the miR-766 target *BAX* and enhanced cell apoptosis [33]. In contrast, our study shows miR-766 is a positive regulator of p53 via directly targeting MDM4, and participates in cell cycle regulation. Interestingly, the *BAX* gene is also a downstream target of p53 [39]. This raises the interesting possibility that a miR-766-p53-Bax axis may exist such that miR-766 maintains p53-mediated cell cycle regulation while concomitantly suppressing Bax-induced cell death. MiR-766 has been shown to be upregulated in colorectal cancer and to be associated with promotion of cell proliferation and anchorage-independent growth by targeting SOX6 in the colorectal adenocarcinoma line SW480 [40]. The results from this study define oncogenic roles to miR-766, which contrasts with our findings. The frequency of p53 mutations in colorectal cancer is over 50%, and the cell line SW480 is p53 mutant. We speculate that miR-766 can function as a tumor suppressor in a wild-type p53 environment but can switch to an oncomiR when p53 is inactivated by mutation.

There are previous reports showing increased miR-766 expression in cancers. MiR-766 was found to be significantly up-regulated in 7 pairs of cutaneous squamous cell carcinoma biopsies [31] and 372 lung adenocarcinoma cases correlating with late stage and poorer prognosis [32]. The status of p53 and MDM4 was not specified in those studies but it is known that the frequency of p53 mutations is high in both cutaneous squamous cell carcinoma (40%-50%) [41] and lung adenocarcinoma (63%) [42]. This knowledge, in combination with our finding that miR-766 is elevated in p53-mutant cancers (Supplementary Figure 1) complicates the association between miR-766 and malignancy identified in the earlier studies of lung adenocarcinoma [32] and cutaneous squamous cell carcinoma [31]. We examined the association between miR-766 and cancer specifically in wild-type p53 tumours of multiple cancer types. While miR-766 was upregulated in breast and liver, it was not significantly changed in stomach cancer and was down-regulated in lung adenocarcinoma (Supplementary Figure 7). With our new findings in mind, the links between miR-766 and malignancy, and hence its previous designation as an oncomiR [33, 40], are ambiguous.

Since miRNAs have multiple targets, their roles in normal biology and disease are highly context-dependent. Indeed, many examples exist where specific miRNAs have dichotomous functions in different tissues or disease states [43]. With this concept in mind, what our study does conclusively indicate is that p53 status will influence the biological functions of miR-766 in tumours. Specifically, miR-766 can target MDM4 and thereby act as a tumor suppressor when MDM4 levels are high, but fails to regulate p53 levels or activity in cells with low expression of MDM4. Thus, in contexts where MDM4 levels are low, or in mutant p53 tumors where the normal transactivation activities of p53 are compromised, miR-766 may potentially exhibit oncogenic behaviour. Having said that, we failed to observe any significant impact on either p53 level or cell proliferation after miR-766 over expression in p53 mutant cell lines (H2009 expressing p53R273L and MDA-MB-468 expressing p53R273H) (Supplementary Figure 6).

MiR-766 was reported to increase in esophageal carcinoma cell lines following 5-fluorouracil treatment [44]. 5-fluorouracil is known to induce the accumulation of p53 and thereby activate p53 dependent pathways [45]. Thus, we speculate that in addition to the known mechanism of p53 stabalization via MDM2 due to its activation of DNA damage pathways, miR-766 is up-regulated and this also contributes to stabilization of p53 via modulation of MDM4. In normal cells, elevated miR-766 levels can provide an additional mechanism that contributes to the stabilization of p53 after external insults. However, in cancer, this function of miR-766 is absent where p53 is mutated or through disruption of other pathways, resulting in miR-766 engaging in alternative functions, some of which may be oncogenic. Based on these results, it is likely that high levels of miR-766 in the context of wild-type p53 may provide an approach for identifying cancers with greater sensitivity to DNA damaging agents.

In summary, our study is the first to identify miR-766 as a novel p53 activator that functions by targeting MDM4 and thereby enhancing the p53 signalling axis. We propose that miR-766 could serve as a potential marker for MDM4 and wild-type p53 based cancer therapies such as MDM4 siRNA and 5-fluorouracil treatment.

## MATERIALS AND METHODS

### Cell lines and reagents

MCF10A, MCF7, HEK293T, MDA-MB468 and U2OS cell lines were purchased from the American Type Culture Collection (ATCC). Other sarcoma cell lines used were supplied by G. Hamilton (University of Vienna, Austria) (TC252) and P. Ambros (St. Anna Children’s Hospital, Austria) (STA-ET-1). SBC-3 and H2009 were kindly given as a gift from Dr. Sandra Hodge (University of Adelaide, Australia). SBC 3 was maintained in DMEM with 10% FBS, HEPES and PSG. H2009 was maintained in RPMI-1640 with 10% FBS. All other cell lines were cultured as previously described [46–48].

### Reverse-transcription PCR (RT-PCR) and real-time RT-PCR

RNA extraction from cells was performed using the RNAeasy mini kit (Qiagen, Valencia, CA USA) and MIReasy mini kit (Qiagen). ND-1000 NanoDrop spectrometer (Thermo Scientific, Wilmington, DE USA) were used to measure RNA concentration. Total RNA and small RNA were reverse-transcribed into cDNA as previously described using Moloney Murine Leukaemia Virus (M-MLV) Reverse Transcriptase (Promega, Madison, WI USA) and random 6’mer primers (Promega) [48].

To determine Real-time PCR reactions were performed using IQ SYBR Green Supermix (BioRad) on a BioRad iCycler (BioRad) as previously described [46]. Primers used for Real-time PCR were listed in Supplementary Table 1. Relative expression levels of specific mRNAs were subsequently determined by the ΔΔCT method and normalized with GAPDH (Primers see Supplementary Table 1).

### Vector construction

For luciferase assay, MDM4 3′UTR parts with miR-766 binding sites were obtained by synthetic single chain oligo annealing (oligo sequences see Supplementary Table 2), and were connected into psiCHECK2 dual-luciferase vector (Promega).

For MDM4 over expression, pENTR223.1 with MDM4 cDNA clone was purchased from Mybiosource. pcDNA3.2-DEST-MDM4 and control were constructed using pcDNA™3.2/GW/D-TOPO® Expression Kit from Raman Sharma (University of Adelaide, Australia) and pENTR223.1-MDM4.

### Transient transfection

Asynchronously growing cells were seeded at 3 × 10^5^ cells/well in six-well plates or 1 × 10^5^ cells/well in 24-well plates. Transfection of cells with 50 nM mimic miR-766 (Genepharma, Shanghai, CN) or 50 nM LNA anti-miR (EXIQON, Denmak) or 100nM siRNA (Sigma Aldrich) was performed using Lipofectamine RNAiMAX (Invitrogen, CA, USA). Transfection of cells with 1ug/mL with psiCHECK2 dual-luciferase reporter vectors or pcDNA3.2-DEST-MDM4 and control was performed using Lipofectamine 2000 (Invitrogen).

### Cell proliferation assay

Cells were plated at 1000 cells/well in 96-well plates 24 hours after transfection and were cultured for 1, 2, 3, 4 and/or 5 days. On the indicated days, cells were incubated with CellTiter-Glo Luminescent Cell Viability Assay Kit (Promega) and fluorescence was measured by LUMIstar Galaxy luminometer (BMG Labtech).

### Cell clonogenic assay

500 cells were placed in 6-well plates 24 hours after transfection, and cultured in complete medium for 10 days (MCF10A and SBC3) and 15 days (U2OS). Colonies were fixed with methanol, stained with 0.1% crystal violet, and counted.

### Soft agar colony formation assay

15000 cells were mixed with 0.35% soft agar and placed in 6-well plates coated with 0.7% soft agar 24 hours after transfection, and cultured for 3 weeks (SBC3) and 5 weeks (U2OS). Colonies were fixed with ethanol, stained with 0.1% crystal violet, and counted.

### Cell cycle analysis

48 and 72 hours after transfections, cells were harvested and fixed as previously described for cell cycle analysis [49]. Fixed cells were stained with 100 μg/mL RNase A (Sigma Aldrich) and 50 μg/mL propidium iodide solution (Sigma Aldrich) in PBS for 45 minutes at 37°C. DNA content was determined by a FACSCalibur™ flow cytometer (BD, CA, USA). Cell cycle profiles were analyzed using WinMDI v2.8 software (Scripps Research Institute).

### Western blot and antibodies

Western blot assay was performed as previously described [48]. Antibodies used include: mouse anti-p53 DO-1 (Santa Cruz Biotechnology, Santa Cruz, CA), Rabbit anti-MDM4 (Bethyl Laboratories), mouse anti-BAX (Invitrogen), mouse anti-a-tublin (Santa Cruz) and mouse anti-β-actin (Sigma Aldrich).

### Luciferase assay

For the luciferase reporter assay, 293T cells were plated in a 24-well plate and then cotransfected with 50 μM of either miR-766 mimic or negative control, and 1 ug of psiCHECK2-MDM4-3′UTR-BS1, psiCHECK2-MDM4-3′UTR-BS2, psiCHECK2-MDM4-3′UTR-BS3, psiCHECK2-MDM4-3′UTR-BS4 or psiCHECK2-MDM4-3′UTR-BS5, using Lipofectamine 2000 (Invitrogen). Cells were collected 48 h after transfection and fluorescence was meassured using the Dual-Luciferase Reporter Assay System (Promega). Both Renilla and Firefly luminescence were measured with a GloMax 20/20 Luminometer (Promega). Renilla luciferase was used as an internal control for any differences in transfection and harvesting efficiencies. Transfections were performed in duplicate and repeated at least three times in independent experiments.

### Gene set enrichment analysis

The Pearson correlation coefficient (r) between miR-766 and 20,501 genes was calculated using sample matched miRNA and mRNA expression profiles from 102 wild-type p53 lung tumors (data was obtained from the TCGA data portal). Genes were subsequently ranked by r value. GSEA Preranked analysis was implemented using the Broad Institute’s public GenePattern server, using default parameters and 3 distinct p53-associated gene sets: the Kannan_TP53_Targets gene sets contain 58 up-regulated and 24 down-regulated p53 gene targets; the Inga_TP53_Targets gene set contains 17 direct p53 targets that were verified to be strongly activated following p53 expression; the Hallmark p53 gene set covers a broader range of both direct and indirect targets of p53.

### Statistical analysis

Data are presented as means ± SD from at least three independent experiments. Student’s t-test was performed using replicate values to compare between groups and indicate significance. Values of p<0.05 were considered statistically significant (as labeled as * in figures), while values of p<0.01 were labeled as ** and p<0,001 as ***.

## SUPPLEMENTARY MATERIALS FIGURES AND TABLES


