# Supplementary material for: MiR-766 induces p53 accumulation and G2/M arrest by directly targeting MDM4
Source: Oncotarget. 2017 Feb 20;8(18):29914–24. doi: 10.18632/oncotarget.15530 (PMC5444713; doi:10.18632/oncotarget.15530)
Supplement: Supplementary file 1 [file oncotarget-08-29914-s001.pdf]

MiR-766 induces p53 accumulation and G2/M arrest by directly targeting MDM4

SUPPLEMENTARY FIGURES AND TABLES

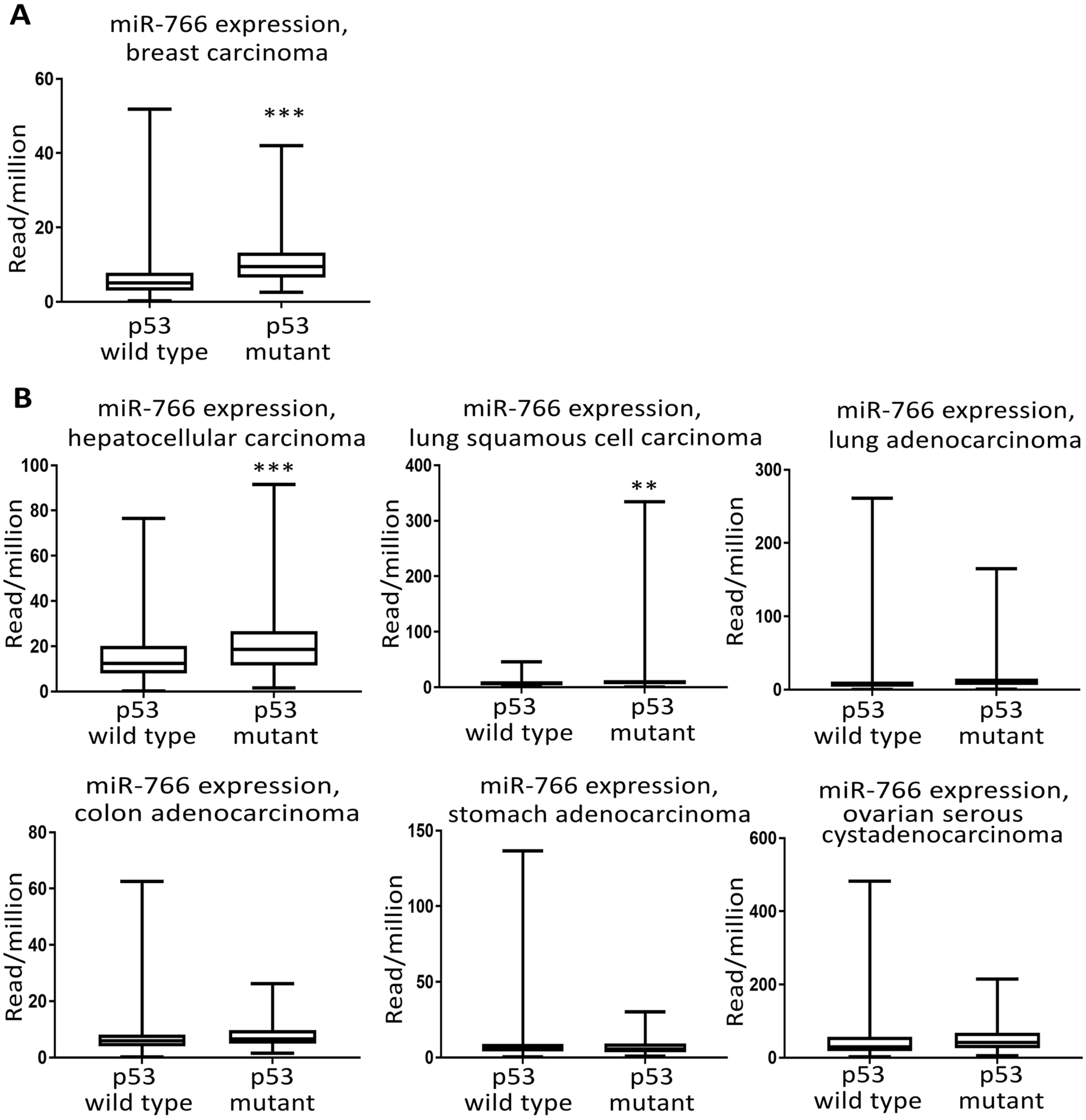

Supplementary Figure 1: (A) Small RNA deep sequencing data of 228 breast cancer tumours expressing wild type p53 and 57 breast cancer tumours expressing mutant p53 were downloaded from TCGA; **(B)** Small RNA deep sequencing data were downloaded from TCGA, including hepatocellular carcinoma (310 expressing wild type p53, 114 expressing mutant p53), lung squamous cell carcinoma (141 expressing wild type p53, 382 expressing mutant p53), lung adenocarcinoma (308 expressing wild type p53, 253 expressing mutant p53), colon adenocarcinoma (337 expressing wild type p53, 70 expressing mutant p53), stomach adenocarcinoma (253 expressing wild type p53, 177 expressing mutant p53) and ovarian serous cystadenocarcinoma (184 expressing wild type p53, 277 expressing mutant p53). (* $p<0.05$, ** $p<0.01$, *** $p<0.001$).

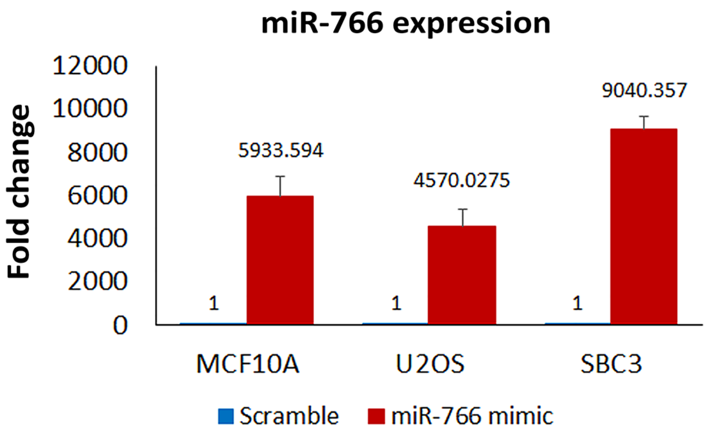

Supplementary Figure 2: MiR-766 over expression in MCF10A, U2OS and SBC3 cell lines with miRNA mimic. Total RNA was collected 24 hours after transfection and miR-766 levels were detected by Taqman real-time PCR. Results were normalized by U6.

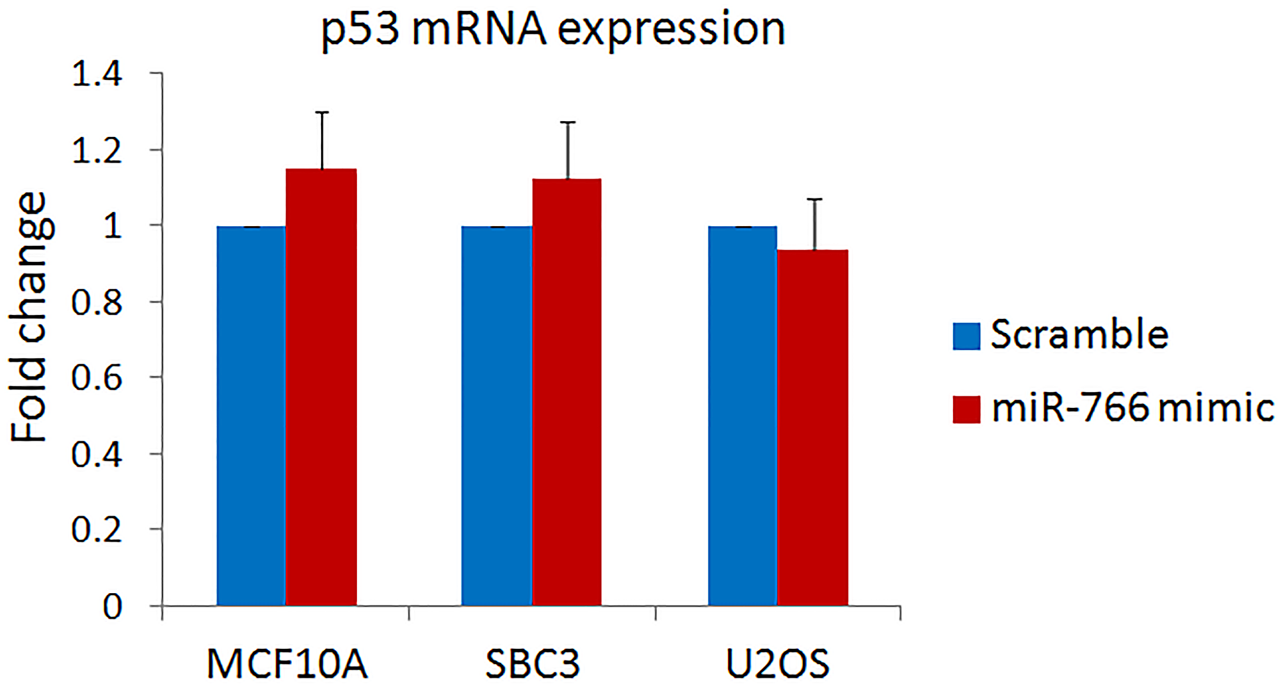

Supplementary Figure 3: MCF10A, SBC3 and U2OS cells were transfected with miR-766 mimic and scramble. Total RNA was collected 24 and 48 hours after transfection and p53 mRNA levels were detected by real-time PCR, results normalized by GAPDH.

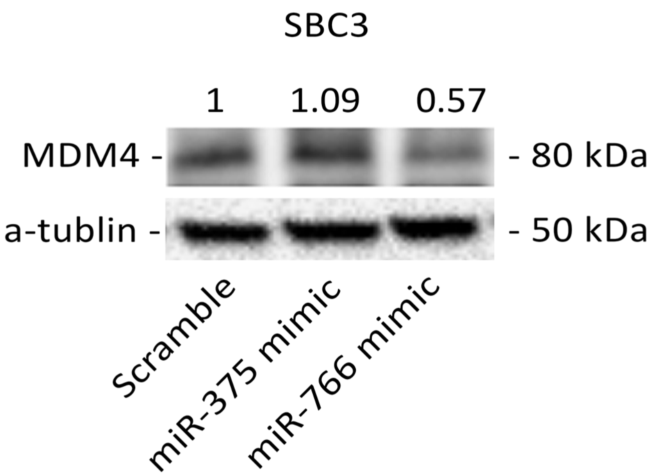

Supplementary Figure 4: SBC3 cells were transfected with miR-375 mimic, miR-766 mimic and scramble as control. Cell lysis was collected 48 hours after transfection and protein levels were detected by western blot. A-tublin was used as loading control.

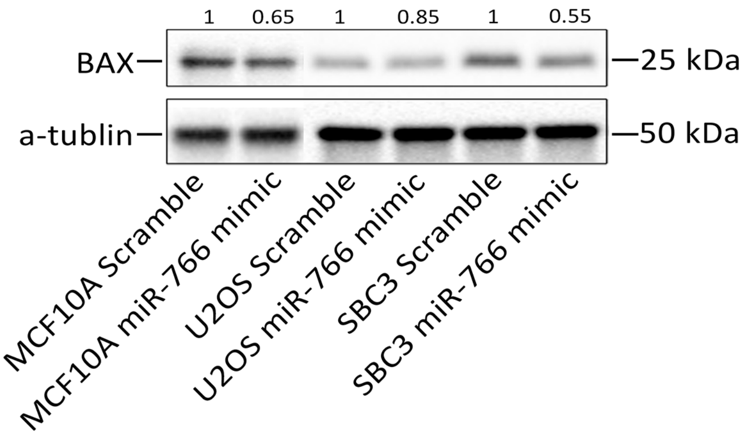

Supplementary Figure 5: MCF10A, U2OS and SBC3 cells were transfected miR-766 mimic and scramble as control. Cell lysis was collected 48 hours after transfection and protein levels were detected by western blot. A-tublin was used as loading control.

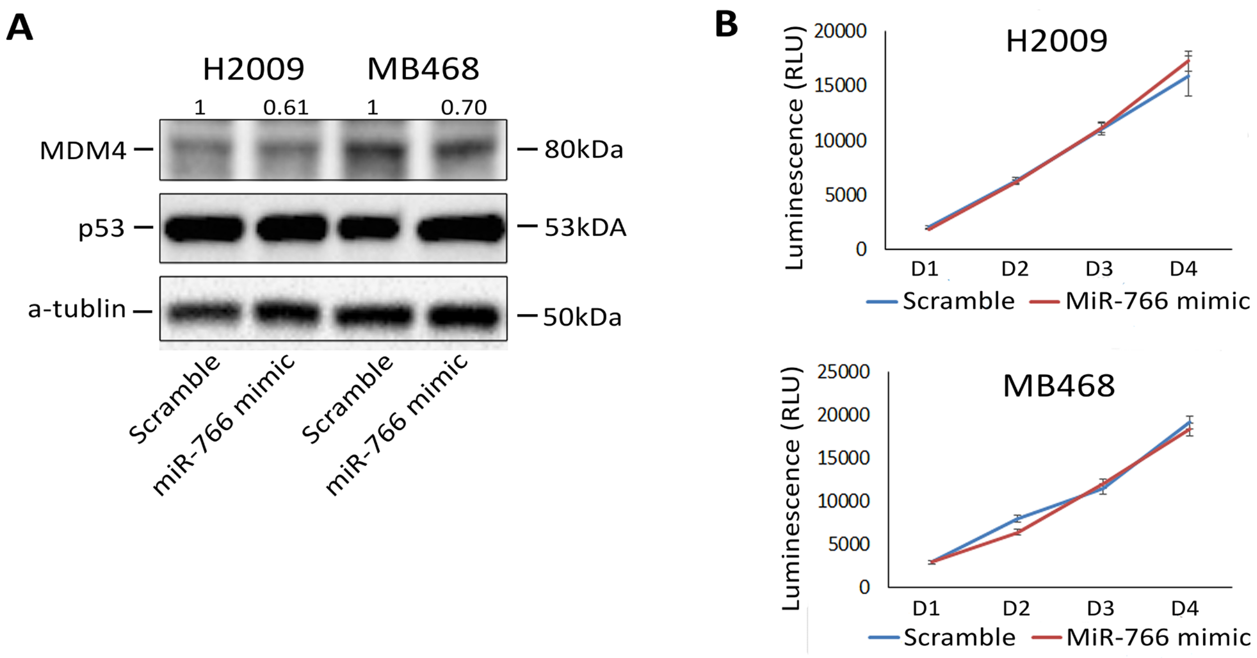

Supplementary Figure 6: H2009 and MDA-MB-468 cells were transfected miR-766 mimic and scramble as control. **(A)** Cell lysis was collected 48 hours after transfection and protein levels were detected by western blot. A-tublin was used as loading control; **(B)** cell proliferation was determined by Cell-TiterGlo Assay from in H2009 and MDA-MB-468 cells.

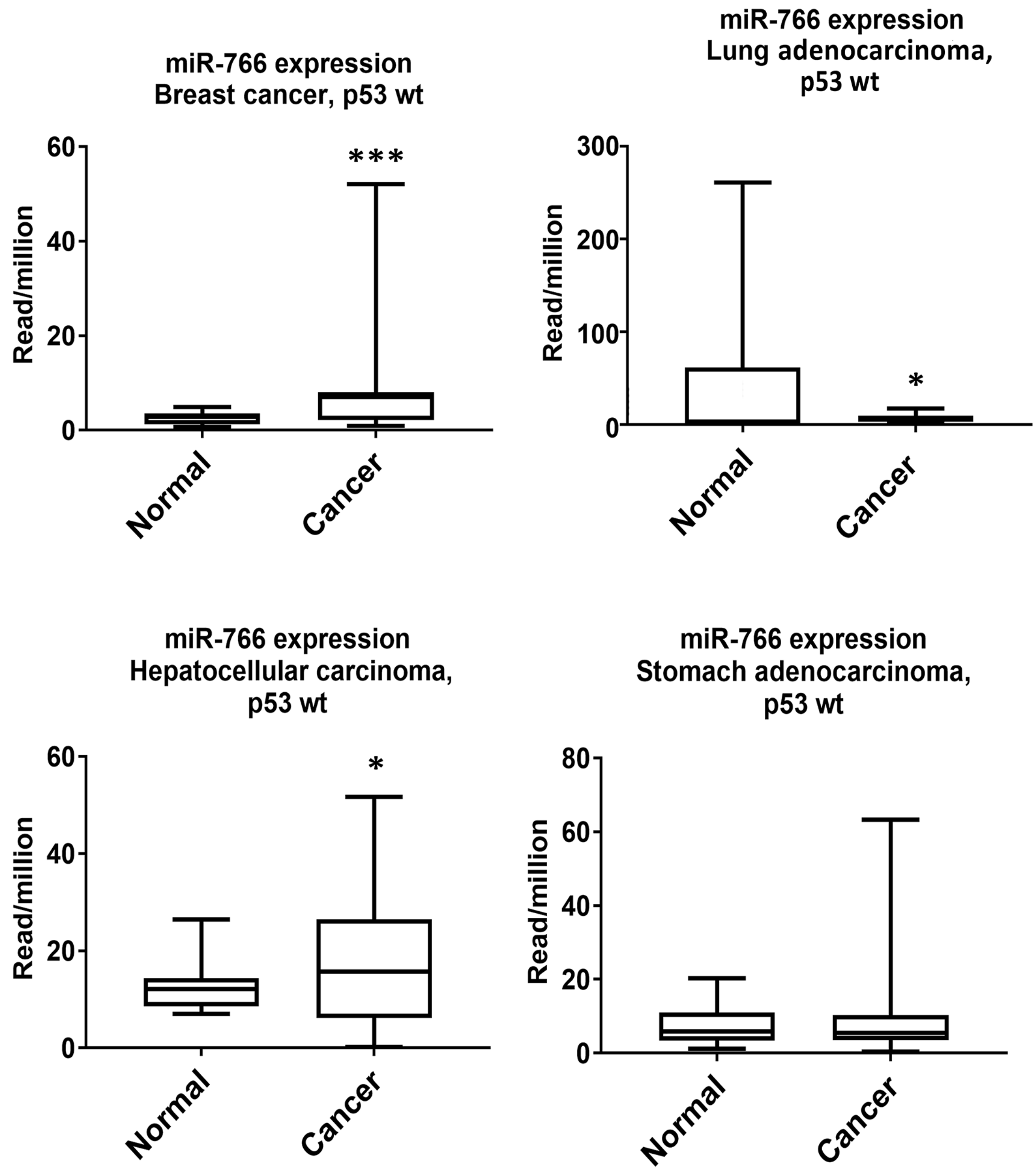

Supplementary Figure 7: (A) Small RNA deep sequencing data of different cancers expressing wild type p53 were downloaded from TCGA and matched with normal tissue from the same patient, including breast cancer (65 patients), lung adenocarcinoma (21 patients), hepatocellular carcinoma (36 patients), and stomach adenocarcinoma (33 patients) (* $p<0.05$, ** $p<0.01$, *** $p<0.001$).

Supplementary Table 1: Primers used for real-time PCR

Gene	Forward (5'-3')	Reverse (5'-3')
MDM4	TCTCGCTCTCGCACAGGATCACA	AACCACCAAGGCAGGCCAGCTA
TP53	GGGCTCCGGGGACACTTTGCG	TGGCAGTGACCCGGAAGGCAGT
GADD45A	AAAGGATGGATAAGGTGGGGG	TGATGTCGTTCTCGCAGCA
SFN	CTGGACAGCCACCTCATCAA	GACGGAAAAGTTCAGGGCCA
GTSE1	GGGAGAAGTTCGTGGAGGTG	ATTCCCTGGGCAAAGCATGA
GAPDH	AGCCTCCCGCTTCGCTCTCTGC	ACCAGGCGCCCAATACGACCAAA

Supplementary Table 2: Oligo sequences for annealing

MiR-766 binding site	Oligo	Sequence
Binding site 1	Forward (5'-3')	TCGAGTGAGAGACGGTCTCACTTTGTCATCCAAGCT GGAGTGCAGTGGTGCAAACACGGCCCACCTCCTGGGCTCGC
	Reverse (5'-3')	GGCCGCGAGCCCAGGAGGTGGGCCGTGTTTGCACCA CTGCACTCCAGCTTGGATGACAAAGTGAGACCGTCTCTCAC
Binding site 2	Forward (5'-3')	TCGAGGACGGTCTCACTTTGTCATCCAAGCTGGA GTGCAGTGGTGCAAACACGGCCCACCTCCTGGGCTCAAGTGGC
	Reverse (5'-3')	GGCCGCCACTTGAGCCCAGGAGGTGGGCCGTGTTTGC ACCACTGCACTCCAGCTTGGATGACAAAGTGAGACCGTCC
Binding site 3	Forward (5'-3')	TCGAGGACAGTCTCACTCTGTTGCCCAGGCTGGAGT GCAATGGCATGATCTCTGCTCACCGCAACCTCTGCCTCCGC
	Reverse (5'-3')	GGCCGCGGAGGCAGAGGTTGCGGTGAGCAGA GATCATGCCATTGCACTCCAGCCTGGGCAACAGAGTGAGACTGTCC
Binding site 4	Forward (5'-3')	TCGAGTACTTGAGATGGAGTTTTGCTCTTGTCG CCCAGGCTGGAGTGCAGTGGAGTGATCTCGGCTCACTGCAACGC
	Reverse (5'-3')	GGCCGCGTTGCAGTGAGCCGAGATCACTCCACTGCA CTCCAGCCTGGGCGACAAGAGCAAAACTCCATCTCAAGTAC
Binding site 5	Forward (5'-3')	TCGAGTAGACGGAGTCTCTCTCTGTCGCCCCGGCT GGAGTGCAGTGGCGCGATCTCGGCTCACTGCAACCTCCGC
	Reverse (5'-3')	GGCCGCGGAGGTTGCAGTGAGCCGAGATCGCGCCAC TGCACTCCAGCCGGGGCGACAGAGAGAGACTCCGTCTAC
